# Clinical Data Mining Related to the Japanese Kampo Concept “*Hie*” (Oversensitivity to Coldness) in Men and Pre- and Postmenopausal Women

**DOI:** 10.1155/2014/832824

**Published:** 2014-02-24

**Authors:** H. Tokunaga, K. Munakata, K. Katayama, R. Yamaguchi, S. Imoto, S. Miyano, K. Watanabe

**Affiliations:** ^1^Center for Kampo Medicine, School of Medicine, Keio University, 35 Shinanomachi, Shinjuku-ku, Tokyo 160-8582, Japan; ^2^Faculty of Environment and Information Study, Keio University, 532 Endo, Fujisawa, Kanagawa 252-0882, Japan; ^3^Laboratory of DNA Information Analysis, Human Genome Center, Institute of Medical Science, University of Tokyo, 4-6-1 Shirokanedai, Minato-ku, Tokyo 108-8639, Japan

## Abstract

“*Hie*” is a subjective oversensitivity to cold and a condition experienced in 60% of Japanese citizens. The condition of *hie* has not been documented in Western medicine. However, in Kampo medicine, *hie* is an important target of treatment, because it has been considered one of the sources of all kinds of diseases. This study aimed to clarify the symptoms and findings associated with *hie* and contribute to increased precision in *hie* diagnosis. During 2005-2006, data from interviews of 1691 patients during their initial visit to the Kampo Clinic of Keio University Hospital were analyzed using a classification and regression tree (CART) analysis, a data mining technique. Symptoms and findings characteristic of each group are follows as, postmenopausal women: fatigability, absence of lower abdominal pain, and absence of hot flashes of feet: women with menstruation: leg swelling, knee pain, and abdominal pain; men: insomnia, leg weakness, and absence of excess saliva. From the perspective of Kampo medicine the result suggested that the feature of *hie* condition in postmenopausal women, women with menstruation, and men is statistically different.

## 1. Introduction

“*Hie*” is a condition that interferes with daily life because a person experiences pain in the entire body or in some parts of the body due to a feeling of cold at temperatures that are not normally considered cold by most people [1]. *Hie* is experienced by about 60% of Japanese people [2, 3]. Most people complaining of oversensitivity to cold temperatures are women. Approximately 45% of these women were within the age range of 15–39 years, whereas 49.6% were within the age range of 40–64.5 years [4]. In addition, men's oversensitivity to cold temperatures has been studied recently and was shown to have approximately one-sixth the frequency of the *hie* experienced by women [5].

Kampo medicine practitioners refer to *hie* as a physical condition in which the hands, the legs, and the lower back rapidly become cold because of poor blood circulation. In general, *hie* is believed to occur because of an abnormality or bias in the balance between heat production/transport and heat dissipation. Peripheral circulatory failure associated with autonomic imbalance is believed to be one of the major causes of *hie*, mainly attributable to stress and an irregular lifestyle. In addition, circulatory insufficiency is likely to occur because of anemia, hypotension, and poor muscular development. People with large amounts of subcutaneous fat are cut off from the temperature in the outside world and are likely to develop peripheral circulatory failure; in other words, such people are likely to develop *hie*. In addition, people with less muscle mass produce less heat and are likely to develop oversensitivity to cold temperatures. A number of other causes of cold oversensitivity have been identified, including decreased levels of estrogen and progesterone. In addition, external factors such as tight underwear, shoes, and socks are likely to cause a circulatory deficit; furthermore, intense temperature differences inside and outside of rooms because of air conditioner usage are likely to cause hypofunctioning of the autonomic nervous system, leading to *hie *[6].

Conversely, symptomatic oversensitivity to cold temperatures is present in Buerger's disease, Raynaud's phenomenon [7, 8], collagenosis, diabetic peripheral neuropathy [9], arteriosclerosis obliterans due to peripheral arterial circulatory insufficiency, and venous circulatory insufficiency, such as in thrombophlebitis, varicose veins, and venous thrombosis, as well as leprosy [10] and reflex sympathetic dystrophy (RSD) [11]. *Hie* is one of the symptoms of hypothyroidism.

Additionally, Western medicine has shown some of the causes of symptomatic *hie*. However, the term *hie* does not exist in Western medicine. Western medicine is currently the mainstream approach for treatment in Japan; in the absence of underlying disease, *hie* is perceived as constitutional. For some people, *hie* is a part of daily life, but most physicians considered it as a condition that does not require active treatment because no objective diagnostic method has yet been established [5].

However, Kampo physicians have regarded *hie* as an important target of medical treatment.

In Kampo medicine, the concept of disease prevention is very strong. An early deviance of the patient's condition should be treated before an obvious pathology has been developed. *hie* is considered as one of such early deviances.

For the treatment of *hie* as a disease entity, prescriptions covered by the health insurance include *shimotsu-to*, *keishi-bukuryogan*, *goshakusan*, and *kami-shoyosan* [12]. This study aimed to educate nonspecialized physicians in Kampo medicine on the importance of treating *hie* and to increase awareness of the utility of Kampo medicine in such treatments.

Therefore, at the Kampo Clinic of Keio University Hospital, the determined therapeutic efficacy and relationship between the symptoms and Kampo medicine-based diagnostic “evidence” were analyzed using the following techniques: data mining based on patients' subjective medical information, Kampo medical diagnoses determined by the physicians, and prescription information. In addition, a system providing support for Kampo medical diagnosis and treatment has been developed for establishing guidelines for general physicians' use, in order to administer Kampo treatment appropriately and for the widespread use of Kampo medicine-based treatment. In this study, the symptoms and findings associated with *hie* were determined on the basis of accumulated patient data during their initial visit and were analyzed for elaborating the diagnosis of *hie*.

## 2. Materials and Methods

The study was conducted on 1691 patients (482 men and 1209 women) whose initial visit at the Kampo Clinic of Keio University Hospital occurred between April 2005 and March 2007. Findings and symptoms associated with oversensitivity to cold temperatures were analyzed using the data mining software, Clementine 12.2 (IBM), on the basis of 137 items including body mass index (BMI); blood pressure; Kampo medical findings (e.g., tongue, pulse, and abdominal examination); and subjective symptoms.

The patients who met any one of the following criteria were considered as *hie* patients: those whose entire body or part of the body displayed symptoms of *hie* and those who were at least “sensitive to cold” or who “easily develop frostbites.” Patients with data deficiencies and those younger than 15 years old were excluded. Patients with atopic dermatitis were excluded because of their particular pathological background. A decision tree analysis (classification and regression tree: CART) was performed separately on the female and male *hie* patients (female: 464/744; men: 102/276; total: 566/1020), using the same software, and the factors related to *hie* were extracted.

In the CART algorithm, we focus on the data of a single variable and divide the patients into two exclusive groups, where the patients in one group have the values that are greater than the cutoff and the values of the patients in the other group are smaller. The variable used for patients' division is selected so that *hie* or non-*hie* patients are more enriched in each of the obtained patient groups than the group before division. We started with the all patients and applied the CART algorithm that generated a tree structure of patients' division, called decision tree, with the variables used. The tree structure can be regarded as a sequential classification of patients and we investigate the sequence of the used variables from a medical viewpoint.

We treated real numerical data such as BMI and blood pressure as categorical variables as much as we possibly could to improve their interpretability, according to the standards of academic societies. The standards are summarized as follows.

- BMI: the patients' BMI were classified into 3 categories on the basis of the standard criteria of the Japan Society for the Study of Obesity: thin (less than 18.5), standard (18.5 or higher but less than 25), and obese (25 or higher).
- Blood pressure: on the basis of the standard criteria of the Japanese Society of Hypertension, the patients were classified into 4 blood pressure categories: optimal, normal, high-normal, and high. In addition, although the academic society has no clear standard criteria stating that *hie* is often encountered in families with hypotension, patients with a systolic blood pressure of less than 100 mmHg were classified into 1 category designated as having “hypotension.” The patients' blood pressure was classified into 5 categories.
- Frequency of urination: on the basis of the standard criteria of respective academic societies, the frequency of urination was classified into 2 categories. A frequency of more than 8 times per day was considered as “frequent daytime urination” (International Continence Society), whereas a urine frequency of once or more per night was considered as “nocturia” (Neurogenic Bladder Society).
- Frequency of stools: although the academic society has no clear-cut standard criteria for this parameter, a stool frequency of once every 3 days or longer was considered as “constipation.”

Furthermore, subjective symptoms found in the women and men were analyzed. In women, *hie* is easy to occur due to decreasing blood levels of estrogen, progesterone [13]. According to the National Livelihood Survey in Japan 1998 by the Ministry of Health and Welfare, women also become aware of *hie* hands and feet, increased rapidly from around the time of postmenopause over the age of 55. Therefore, we thought that the properties of *hie* are different in the presence or absence of menstruation. After the overall analysis was performed, the findings in postmenopausal female patients and in women who menstruated were compared (Table 1). The analysis procedure was as follows.

1. A two-branch decision tree was constructed using CART to classify the data on the basis of the existence or absence of *hie*.
2. Based on each decision tree, we performed a knowledge extraction on the following items:
  - significance test of the presence or absence of *hie* in each population after divergence;
  - Kampo-based interpretation of each branch (Table 2);
  - test of the significance of biases in the findings of each population after divergence.
3. Comparison of findings.

CART is a data mining method that allows the classification and regression of samples with multiple, measurable features. Our study method utilized the presence or absence of *hie* as a patient information label and used the patient interview data as feature variables to repeatedly divide the patient population into 2 exclusive groups based on the patient population size (i.e., large or small, if the feature value was a continuous variable); assignment to a specific category (if the feature variable was a categorical variable, e.g., blood pressure); and rate of *hie* (e.g., high and low rates). This method allowed for statistical learning based on multiple feature variables, as well as the generation of a decision tree. Significant differences in (2) (A) and (2) (C) were examined using Fisher's exact test to compare the original population (or “base population,” namely, the population at the highest level, which was the root node of the decision tree) and the populations after the division. We call this type of analysis subanalysis.

## 3. Results

### 3.1. Postmenopausal Women

**Figure 1:**
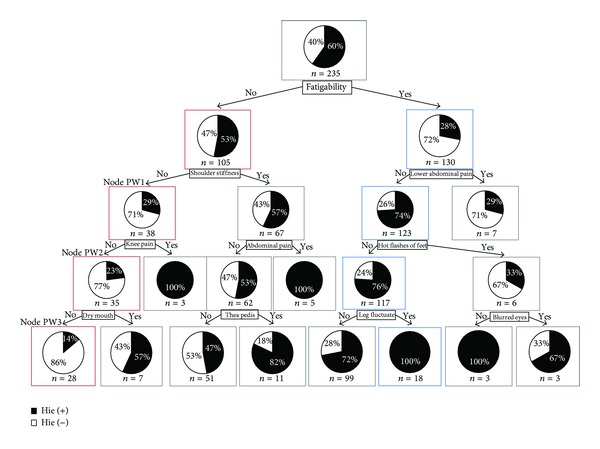
CART of postmenopausal women by *hie* related symptoms and findings. The root node of Figure 1 indicates that there were 235 total observations (*n* = 235), and the black part of the pie chart means 60% of “*n,*” who have had an episode of *hie* (hie(+)). And the white part of the pie chart means 40% of “*n,*” who have not had an episode of *hie* (hie(−)). Each node below the root node has the same meaning.

First, a decision tree pertaining to the postmenopausal women group was constructed using CART (Figure 1). Fisher's exact test was performed to detect populations (nodes) with significantly larger (or smaller) numbers of patients with *hie*, as compared to that of the base population (node 0). Originally, 60% of the patients in the base population presented with *hie*.

#### 3.1.1. Group of Patients with Significantly More *Hie*

The result showed that a path formed a population with significantly more *hie* on the right side of the decision tree than on the left side; in Figure 1, the variables surrounded by blue boxes are included in this path. This path included patients who had the following characteristics: fatigability (+), lower abdominal pain (−), hot flashes of feet (−), and leg fluctuate (−); all patients in the resulting group had *hie*.

#### 3.1.2. Group of Patients with Significantly Less *Hie*

On the other hand, in Figure 1, on the left side of the decision tree, a path with red-boxed variables gathers a population with a significantly less rate of *hie*. The patients selected by this path had the following characteristics: fatigability (−), shoulder stiffness (−), knee pain (−), and dry mouth (−); only 14% of the patients in the resulting group had *hie*.

Next, the significance of the bias in the findings from the nodes with significant differences in *hie* was examined using Fisher's exact test. None of the nodes in the path with a significantly more rate of *hie* showed any finding of bias. Conversely, in the path with significantly less *hie*, we found several findings.

- In the node PW1, the number of patients whose excess of pulse are greater than those with deficiency of pulse (excess > deficiency), while in the base population we found that patients with deficiency of pulse are greater than those with excess of pulse (deficiency > excess).
- In the nodes PW1 and PW2, the tenderness points of blood stasis were found to be 13.6% and 11.4% which were approximately 18% and 20% lower than that of the result found in the base population (31.5%), respectively.
- In the node PW3, the number of patients whose humidity of tongue is dry is almost equal to those with normal humidity of tongue and is greater than those with wet (dry = normal > wet), while in the base population we found that patients with normal humidity of tongue are greater than those with dry and dry is much greater than wet (normal > dry ≫ wet).
- In the node PW3, the number of patients whose size of tongue are normal is greater than those with enlarge size of tongue and enlarge is greater than thin (normal > enlarge > thin). On the other hand, in the base population we found that patients with normal size of tongue are much greater than enlarge size of tongue and enlarge is greater than thin (normal ≫ enlarge > thin).
- In the node PW3, the tenderness point of blood stasis was found to be 10.7%, which was 20% less than that of the result found in the base population.

We summarized the results of subanalysis in Table 3.

### 3.2. Women with Menstruation

**Figure 2:**
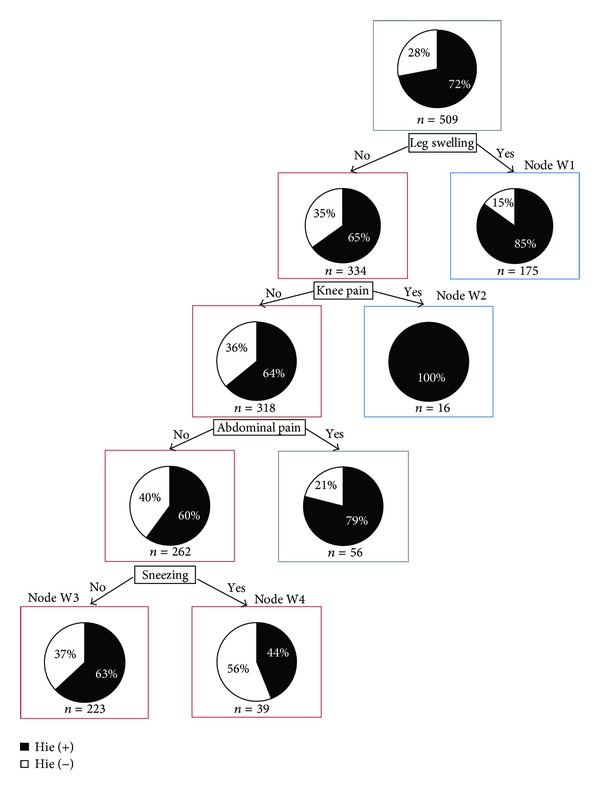
CART of women with menstruation by *hie* related symptoms and findings. The root node of Figure 2 indicates that there were 509 total observations (*n* = 509), and the black part of the pie chart means 72% of “*n,*” who have had an episode of *hie* (hie(+)). And the white part of the pie chart means 28% of “*n,*” who have not had an episode of *hie* (hie(−)). Each node below the root node has the same meaning.

Second, decision tree pertaining to the women with menstruation was constructed (Figure 2). Paths with significantly more (or less) rates of *hie* were searched through comparison with the base population (node 0) using Fisher's exact test. Originally, 72% of the patients in the base population presented with *hie*. Although we described 2 paths with significantly less rates of *hie*, they were counted as one single path because most of their nodes were common to both.

#### 3.2.1. Group of Patients with Significantly More *Hie*

In Figure 2, on the right side of the decision tree, we found 2 paths indicated by the blue-boxed variables. These paths comprised a population with significantly more rates of *hie*. The patients selected by one path had leg swelling (+), and 85% of the patients in the resulting group had *hie*. The other path had leg swelling (−) and knee pain (+), and all patients in the resulting group had *hie*.

#### 3.2.2. Group of Patients with Significantly Less *Hie*

In Figure 2, on the left side of the decision tree, we found a path indicated by the connections of red-boxed nodes (more strictly there are 2 paths, but almost all nodes are shared and we consider the connection of red-boxed nodes as one path). The patients in the terminal nodes (nodes W3 and W4) had leg swelling (−), knee pain (−), abdominal pain (−), and sneezing (−) or (+) and this path formed a population with significantly less rates of *hie* (63% of the patients in the node W3 had *hie* and 44% of the patients in the node W4 had* hie*).

The bias of the findings in each node was examined using Fisher's exact test. In the nodes with a significantly more *hie* rate, we found the following.

- From BMI of the patients in the node W1, the percentage of the patients with thin body types was significantly more than that in the base population (*P* = 0.084).
- In the node W2, there are no patients with hypotension or normal blood pressure, while in the base population they are approximately 22% for hypotension and 10% for normal blood pressure. Also, in the base population, while the number of patients with high blood pressure is greater than that of the patients with high-normal, in the node W2 they are switched.

In the path with less rates of *hie*, namely, in terms of abdominal strength, the following group was determined.

- In the node W3, excess of abdominal strength accounted for 2.7% of the cases, which was weakly significantly less than that of the observed result of 4.9% in the base population (*P* = 0.062).
- In the node W4, the presence of epigastric tightness and resistance accounted for 0%, which was weakly significantly less than that of the observed result of 9.6% in the base population (*P* = 0.064).

### 3.3. Men

**Figure 3:**
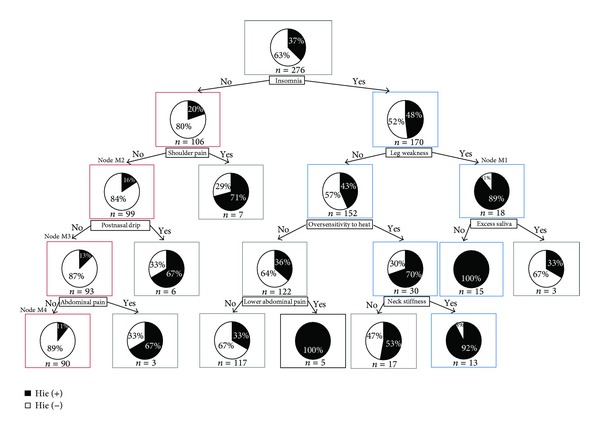
CART of men by *hie* related symptoms and findings. The root node of Figure 3 indicates that there were 276 total observations (*n* = 276), and the black part of the pie chart means 37% of “*n,*” who have had an episode of *hie* (hie(+)). And the white part of the pie chart means 63% of “*n,*” who have not had an episode of *hie* (hie(−)). Each node below the root node has the same meaning.

Finally, a decision tree pertaining to *hie* in men was constructed (Figure 3). Paths that had significant differences in *hie* were searched using Fisher's exact test and by comparing this with the base population (node 0). The findings showed 2 paths with significantly more *hie* rates and one path with significantly less *hie* rates.

#### 3.3.1. Group of Patients with Significantly More *Hie*

In Figure 3, on the right side of the decision tree, we found 2 paths indicated by the connection of blue-boxed nodes. These paths formed a population with significantly more rates of *hie*. All patients in the terminal node of the 1st path, which is characterized by insomnia (+), leg weakness (+), and excess saliva (−), had *hie* and in the terminal node of the 2nd path, with insomnia (+), leg weakness (−), oversensitivity to heat (+), and neck stiffness (+), 92% of the patients had *hie*.

#### 3.3.2. Group of Patients with Significantly Less *Hie*

In Figure 3, on the left side of the decision tree, we found a path indicated by the red-boxed nodes. This path formed a population with significantly less rates of *hie*. The patients in the terminal node of this path had insomnia (−), shoulder pain (−), postnasal drip (−), and abdominal pain (−), and only 11% of patients in the terminal node had *hie*.

Next, bias in the findings in each node was examined using Fisher's exact test. The results can be summarized as follows.

- In the 1st path described above, in which the rates of *hie* were high, namely, in the node M1, abnormal complexion accounted for 46.2% of the cases, which was significantly more than that of the observed result of 15.2% in the base population (*P* = 0.012).
- The findings in the 2nd path showed no significant bias.
- In the path with less *hie *shown by the red-boxed nodes, we found the following about pulse depth. In the nodes M2, M3, and M4, a floating pulse in these groups accounted for 0%, which was significantly less than that of the observed result of 11.6% in the base population (*P* = 0.013 for node M2, *P* = 0.023 for node M3, and *P* = 0.023 for node M4).
- In the node M4, the presence of epigastric stiffness accounted for 0%, which was significantly less than that of the observed result of 0.72% in the base population (*P* = 0.013).

## 4. Discussions

In the past, treatment with Kampo medicine was performed based on experience; however, today, medical treatments are based on data. Nevertheless, *hie* has not yet been studied in a large number of patients.

Previous studies on *hie* have focused on comparing single variables that may have influenced the development of subjective symptoms among healthy subjects and diagnosed patients [14]. This current study serves as the first effort to analyze *hie* in a large number of patients using multivariate data mining and statistical analysis methods and to highlight its properties on the basis of strongly related symptoms and their combinations. The results of this study suggested that the symptoms strongly related to *hie* were different among the groups of postmenopausal women, women with menstruation, and men and that the percentages of *hie* were different depending on the symptom combinations. By interpreting these symptoms from the perspective of Kampo medicine, common and differing traits for *hie* in each group were elucidated.

With respect to the findings pertaining to the postmenopausal women with *hie*, from the Kampo medicine perspective, the previously mentioned results suggested that the blood stasis pattern and the Qi deficiency pattern were important in the presence or absence of *hie* after menopause.

From the perspective of Kampo medicine, the above-mentioned results pertaining to the presence or absence of *hie* in women with a normal menstrual cycle suggested that a blood stasis and water retention pattern played an important role in the development of the *hie* condition. In the nodes with a large number of patients with *hie*, the findings were characterized by the unexpected fact that both hypotension and normal blood pressure accounted for 0%, despite the initial expectation for a high percentage.

As for the summary of the findings pertaining to male patients with *hie*, the results suggest that from the perspective of Kampo medicine a Qi stagnation pattern, a deficiency of lower energizer pattern, and a blood stasis pattern influenced the development of the *hie* condition.

Interestingly, “oversensitivity to heat (+)” was included despite the fact that the node was comprised of a large number of patients with *hie*. *Hie* in this node could probably be attributable to autonomic imbalance.

From this rationale, it became clear that *hie* and a blood stasis pattern are both concepts of Kampo medicine and are closely related. Most prescriptions used to cure *hie* actually treat a blood stasis pattern as well. Moreover, *hie* is closely related to the peripheral circulation disorder. From the viewpoint of the peripheral circulatory failure, the pathology of *hie* is estimated to be related to the following: a decrease in cardiac output, functional and organic changes in the vessel wall [15], and changes in blood fluidity [16, 17], as well as changes in platelet function, coagulation, and fibrinolysis [18]. From the viewpoint of Western medicine, attempts to clarify the factors involved in a blood stasis pattern have been made.

At our Kampo clinic, our actual experience has shown marked differences between women who menstruate and postmenopausal women, as well as between men and women. Thus, our results statistically confirm what Kampo doctors had already collected during their long clinical experience with respect to the concept *hie*. The differences in the *hie* characteristics in each group can be considered to be influenced by differences in the hormonal environment and age-related changes and gender, as well as other factors.

Our research group will conduct further studies using a larger number of patients, include symptoms other than *hie*, and involve a time-course analysis of the efficacy of the prescribed treatment. We hope this study will contribute to creating more transparency and evidence in the field of traditional Japanese Kampo medicine.

## 5. Conclusion

By using the method of data mining, our study helped to statistically clarify that the characteristics of the concept *hie* are different among postmenopausal women, menstruating women, and men. Thus, the findings suggested from experience could be confirmed. The results from this study may also support physicians in selecting the appropriate Kampo prescription.

## Figures and Tables

**Table 1:** Demographic background of patients (Av. ± SD).

	Total (*n* = 1020)	Postmenopause (*n* = 235)	Menstruating (*n* = 509)	Male (*n* = 276)
Age (years)	47.3 ± 17.1	62.4 ± 9.8	36.1 ± 9.6	52.1 ± 18.3
Height (cm)	159.8 ± 7.8	154.2 ± 5.7	158.9 ± 5.6	168.1 ± 6.2
Weight (kg)	55.0 ± 11.0	51.3 ± 9.4	52.6 ± 8.7	64.5 ± 11.1
Body mass index (kg/m^2^)	21.5 ± 3.5	21.6 ± 3.6	20.9 ± 3.3	22.8 ± 3.4
Systolic blood pressure (mmHg)	116.9 ± 19.1	126.3 ± 18.7	109.0 ± 15.7	122.9 ± 19.1
Diastolic blood pressure (mmHg)	71.3 ± 13.3	74.8 ± 12.7	67.2 ± 11.7	76.7 ± 14.1
Percentage of *hie *symptom (%)	59.0	60.0	72.0	37.0
Percentage of frequent urination (%)	32.0	35.7	33.0	24.0
Percentage of constipation (%)	7.8	5.5	11.4	4.7

**Table 2:** Selected Kampo terms (ICD11Beta).

Kampo terms	ICD terms	Description
*Kyo *	Deficiency pattern	A pattern characterized by fatigue or weakness; at the onset of febrile condition, characterized by cold sensitivity and tendency to sweat, floating weak pulse; in case of nonfebrile condition, characterized by weak pulse, weak abdominal wall. It may be explained by weak response to pathogens.

*Jitsu *	Excess pattern	A pattern characterized by (at the onset of febrile condition) severe chills with no sweating, strong pulse; in case of nonfebrile condition, strong pulse, strong abdominal wall. It may be explained by strong response to pathogens.

*Oketsu *	Blood stasis patterns	A pattern characterized by various menstrual disorders such as amenorrhea, dysmenorrhea, and menopausal syndrome; lower abdominal fullness, varicose veins, hemorrhoids, and mood swings. It may be explained by impaired peripheral blood circulation or obstruction of venous return.

*Suidoku *	Fluid disturbance patterns	A pattern characterized by fluid retention or dehydration in the digestive tract, body tissues, or cavities. It may be explained by abnormal distribution of body fluids and an imbalance in fluids and electrolytes.

*Kikyo *	Qi deficiency pattern	A pattern characterized by decreased vitality, fatigue, weakness, and appetite loss. It may be explained by deficiency of energy, such as exhausted state, which is almost always accompanied by deficiency of the upper abdominal region.

*Kiutsu/Kitai *	Qi stagnation pattern	A pattern characterized by feeling of obstruction at the level of throat, feeling of ear tube obstruction, abdominal distension due to intestinal gas retention, depressive state, and intractable pain. It may be explained by functional discommunication leading to gas retention in bowels, mental depression, or other manifestations.

*Geshou no kyo *	Deficiency in lower energizer pattern	A pattern characterized by dysfunctions of the urogenital system. It may be explained by weakness in the lower part of the body.

**Table 3:** Summary of subanalysis: findings and *P* value.

		Findings	*P* value
Node	PW1	Tenderness point of blood stasis	0.080
		Excess of pulse	0.094
	PW2	Tenderness point of blood stasis	0.065
	PW3	Tenderness point of blood stasis	0.075
		Humidity of tongue	0.097
		Size of tongue	0.044
	W1	BMI < 18.5	0.084
	W2	Blood pressure	0.016
	W3	Excess of abdominal strength	0.062
	W4	Epigastric tightness and resistance	0.064
	M1	Abnormal complexion	0.012
	M2	Floating pulse	0.013
	M3	Floating pulse	0.023
	M4	Floating pulse	0.023
		Epigastric stiffness	0.013
